# Lipid metabolism transcriptomics of murine microglia in Alzheimer’s disease and neuroinflammation

**DOI:** 10.1038/s41598-023-41897-6

**Published:** 2023-09-08

**Authors:** Daniel C. Shippy, Tyler K. Ulland

**Affiliations:** Department of Pathology and Laboratory Medicine, University of Wisconsin, Madison, WI USA

**Keywords:** Neuroimmunology, Immunology

## Abstract

Alzheimer’s disease (AD) is a neurodegenerative disorder characterized by the accumulation of amyloid-β (Aβ) plaques followed by intracellular neurofibrillary tangles (NFTs) composed of hyperphosphorylated tau. An unrestrained immune response by microglia, the resident cells of the central nervous system (CNS), leads to neuroinflammation which can amplify AD pathology. AD pathology is also driven by metabolic dysfunction with strong correlations between dementia and metabolic disorders such as diabetes, hypercholesterolemia, and hypertriglyceridemia. Since elevated cholesterol and triglyceride levels appear to be a major risk factor for developing AD, we investigated the lipid metabolism transcriptome in an AD versus non-AD state using RNA-sequencing (RNA-seq) and microarray datasets from N9 cells and murine microglia. We identified 52 differentially expressed genes (DEG) linked to lipid metabolism in LPS-stimulated N9 microglia versus unstimulated control cells using RNA-seq, 86 lipid metabolism DEG in 5XFAD versus wild-type mice by microarray, with 16 DEG common between both datasets. Functional enrichment and network analyses identified several biological processes and molecular functions, such as cholesterol homeostasis, insulin signaling, and triglyceride metabolism. Furthermore, therapeutic drugs targeting lipid metabolism DEG found in our study were identified. Focusing on drugs that target genes associated with lipid metabolism and neuroinflammation could provide new targets for AD drug development.

## Introduction

Alzheimer’s disease (AD) is a neurodegenerative disorder characterized by the accumulation of amyloid-β (Aβ) plaques followed by intracellular neurofibrillary tangles (NFTs) composed of hyperphosphorylated tau^1^. Microglia, the innate immune cells of the central nervous system (CNS), facilitate Aβ and tau clearance, but also promote neuroinflammation that damages neurons and exacerbates AD pathology^2^. To date, few effective treatments for AD exist, and most AD drug research is focused on Aβ and tau reduction. Recently, the FDA approved the anti-amyloid antibodies aducanumab and lecanemab for the treatment of AD^3,4^. Although both antibodies reduce the rate of cognitive decline, legitimate questions remain regarding the efficacy and safety of aducanumab and lecanemab^5,6^. Furthermore, since only Aβ is targeted, and as AD is a complex disease, it is estimated that only 8–20% of patients with AD will be eligible for treatment^7^, so there is still an urgent need for new therapeutic interventions for AD. Recent studies suggest AD neuropathology is driven by metabolic dysfunction with strong correlations between dementia and metabolic disorders such as hypertension, diabetes, hypercholesterolemia, and hypertriglyceridemia^8^. Furthermore, increased microglial lipid metabolism provides energy for microglial activation and effector functions, and alterations in microglial lipid metabolism are implicated in the development of several neurological disorders, including AD^9,10^. Therefore, the characterization of metabolic networks, and identification of drugs targeting the genes in these networks, could be a potential treatment strategy for AD.

Lipids account for most of the dry mass of the brain^11^ and lipid metabolism changes during the aging process^12^. Lipids in the brain can largely be classified as sterols, fatty acids, phospholipids, glycerolipids, and sphingolipids^13^. The human brain has the highest level of cholesterol compared to other organs and is a vital component of eukaryotic membranes^14^. Apolipoprotein E (ApoE) is the main cholesterol carrier in the brain^15^, and is considered the strongest genetic risk factor for the development of late-onset AD^16^. Individuals with one copy of the *apoE* ε4 allele increase their risk of developing AD approximately fourfold, while individuals with two copies increase their risk by approximately 12-fold^16^. Additionally, *apoE* ε4 allele carriers are more likely to develop hypercholesterolemia and hypertriglyceridemia^17–19^. A recent study suggests management of blood glucose and cholesterol levels in early adulthood has a significant impact on AD risk later in life^20^. Based on these findings, limiting dysfunctions in lipid metabolism could be vital for the prevention of AD, particularly for *apoE* ε4 allele carriers.

Since metabolic disorders appear to be a major risk factor for developing AD, we investigated the lipid metabolism transcriptome in an AD versus non-AD state using RNA-sequencing (RNA-seq) and microarray datasets from N9 and murine microglia. We identified 52 lipid metabolism differentially expressed genes (DEG) in LPS-stimulated N9 microglia versus unstimulated control cells using RNA-seq, 86 lipid metabolism DEG in 5XFAD versus wild-type mice by microarray, with 16 DEG common between both datasets. Functional enrichment and network analyses identified several biological processes and molecular functions, such as cholesterol homeostasis, insulin signaling, and triglyceride metabolism as being dysregulated in neuroinflammation and AD. Furthermore, gene–drug interactions were characterized to identify drugs targeting lipid metabolism genes. Overall, these data show the lipid metabolism transcriptional response by murine microglia during AD progression with the potential for new treatment strategies directed towards AD.

## Results

### AD promotes differential expression of lipid metabolism genes

**Figure 1:**
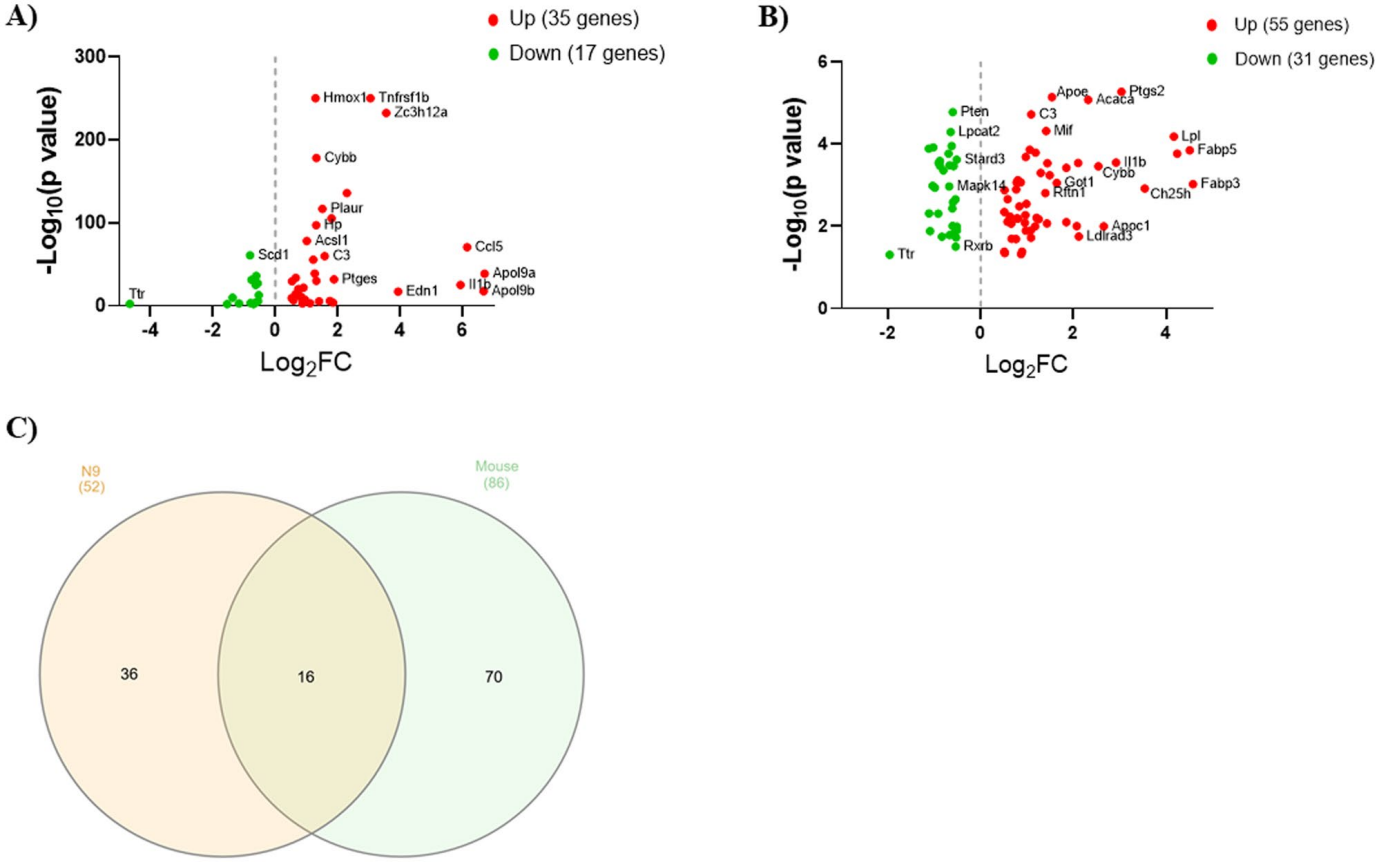
Differentially expressed lipid metabolism genes in AD. **(A)** Scatter plot of lipid metabolism DEG (log_2_FC > 0.5, FDR-adjusted *P*-value < 0.05) by RNA-seq in N9 microglia stimulated with LPS (1 µg/ml) for 6 h versus unstimulated control cells. **(B)** Scatter plot of lipid metabolism DEG (log_2_FC > 0.5, *P* < 0.05) by microarray in microglia isolated from the brains of 5XFAD mice versus wild-type mice (8 months old). For both scatter plots, up-regulated genes are shown in red and down-regulated genes are shown in green. Data are graphed as log_2_FC versus − log_10_ (*P*-value). **(C)** Venn diagram demonstrating overlap in lipid metabolism DEG between the N9 and mouse microglia datasets.

In order to identify lipid metabolism genes involved in AD-associated neuroinflammation, we analyzed gene expression datasets from LPS-stimulated N9 microglia and microglia isolated from 8-month-old 5XFAD mice. Analysis of RNA-seq data from LPS-stimulated N9 microglia versus non-stimulated control cells revealed a total of 52 lipid metabolism DEG (log_2_FC > 0.5, FDR-adjusted *p*-value < 0.05). Of these 52 DEG, 35 were up-regulated and 17 were down-regulated (Fig. 1A). Apolipoprotein L 9a (*Apol9a*) was the most up-regulated lipid metabolism gene (log_2_FC = 6.71) and transthyretin (*Ttr*) was the most down-regulated lipid metabolism gene (log_2_FC =  − 4.66) (Supplementary Table S1).

We identified 86 lipid metabolism DEG (log_2_FC > 0.5, *p* < 0.05) in publicly available transcriptional data from sorted microglia from female 8-month-old 5XFAD mice versus wild-type mice^21^. The 5XFAD mouse model of AD rapidly develops severe amyloid pathology with Aβ plaque accumulation beginning around 2 months of age^22^. Of the 86 lipid metabolism DEG, 55 were up-regulated and 31 were down-regulated (Fig. 1B). Fatty acid binding protein 3 (*Fabp3*) was the most up-regulated lipid metabolism gene (log_2_FC = 4.58) and transthyretin (*Ttr*) was the most down-regulated lipid metabolism gene (log_2_FC =  − 1.97) (Supplementary Table S2).

In total, 16 lipid metabolism DEG overlapped between the datasets (Fig. 1C). A complete list of the 16 lipid metabolism genes and their fold change values for both datasets is shown in Table 1.

**Table 1:** Altered lipid metabolism genes common to both datasets.

Gene	ID^1^	Description	N9 Log_2_FC	Mouse Log_2_FC
*Il1b*	27398	Interleukin 1 beta	5.94	2.92
*Zc3h12a*	42677	Zinc finger CCCH type containing 12A	3.56	0.67
*Olr1*	30162	Oxidized low density lipoprotein (lectin-like) receptor 1	1.75	1.43
*C3*	24164	Complement component 3	1.59	1.09
*Plaur*	46223	Plasminogen activator, urokinase receptor	1.51	1.85
*Cybb*	15340	Cytochrome b-245, beta polypeptide	1.31	2.54
*Ptgs2*	32487	Prostaglandin-endoperoxide synthase 2	1.22	3.03
*Cxcl16*	18920	Chemokine (C-X-C motif) ligand 16	1.11	1.18
*Plscr1*	32369	Phospholipid scramblase 1	0.90	0.98
*Fabp3*	28773	Fatty acid binding protein 3, muscle and heart	0.74	4.58
*Stard3*	18167	START domain containing 3	0.52	− 0.52
*Tlr4*	39005	Toll-like receptor 4	− 0.53	− 0.99
*Pik3cd*	39936	Phosphatidylinositol-4,5-bisphosphate 3-kinase catalytic subunit delta	− 0.63	− 0.59
*Apoe*	02985	Apolipoprotein E	− 0.68	1.54
*Fdps*	59743	Farnesyl diphosphate synthetase	− 0.79	1.25
*Ttr*	61808	Transthyretin	− 4.66	− 1.97

^1^All gene IDs start with ENSMUSG000000.

### Pathway and enrichment analysis of altered lipid metabolism genes

**Figure 2:**
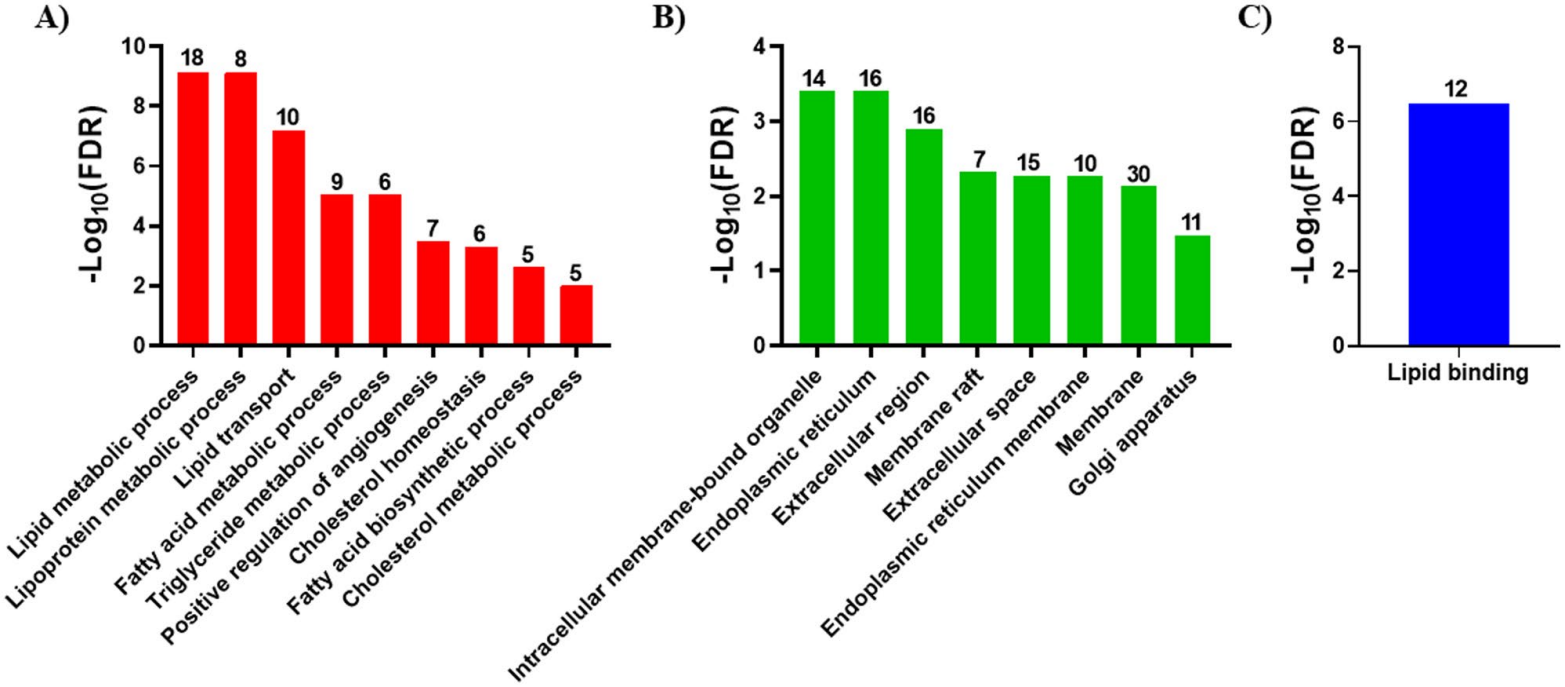
N9 microglia GO enrichment analysis. Biological function analyses for the N9 microglial lipid metabolism DEG was performed using DAVID. Analyses were performed for biological process (BP) (**A**), cellular component (CC) (**B**), and molecular function (MF) (**C**). Pathways are shown in descending order based on − log_10_ FDR. The number of genes associated with each GO term is shown above each bar. Only GO terms with a gene count ≥ 5 and FDR < 0.05 were considered significant.

Gene ontology (GO), Kyoto Encyclopedia of Genes and Genomes (KEGG), and Search Tool for the Retrieval of Interacting Genes/Proteins (STRING) analyses were performed on the N9 and mouse microglial lipid metabolism DEG. For N9 microglia, Biological Process (BP) GO indicated the DEG participated in lipid metabolic process, lipoprotein metabolic process, lipid transport, fatty acid metabolic process, triglyceride metabolic process, positive regulation of angiogenesis, cholesterol homeostasis, fatty acid biosynthetic process, and cholesterol metabolic process (Fig. 2A). Cellular Component (CC) GO indicated the N9 microglial lipid metabolism DEG were located in the intracellular membrane-bounded organelle, endoplasmic reticulum, extracellular region, membrane raft, extracellular space, endoplasmic reticulum membrane, membrane, and Golgi apparatus (Fig. 2B). Molecular Function (MF) GO indicated the N9 microglial lipid metabolism DEG were involved in lipid binding (Fig. 2C). A complete list of the GO, false discovery rates, and genes associated with each GO is shown in Supplementary Table S3.

**Figure 3:**
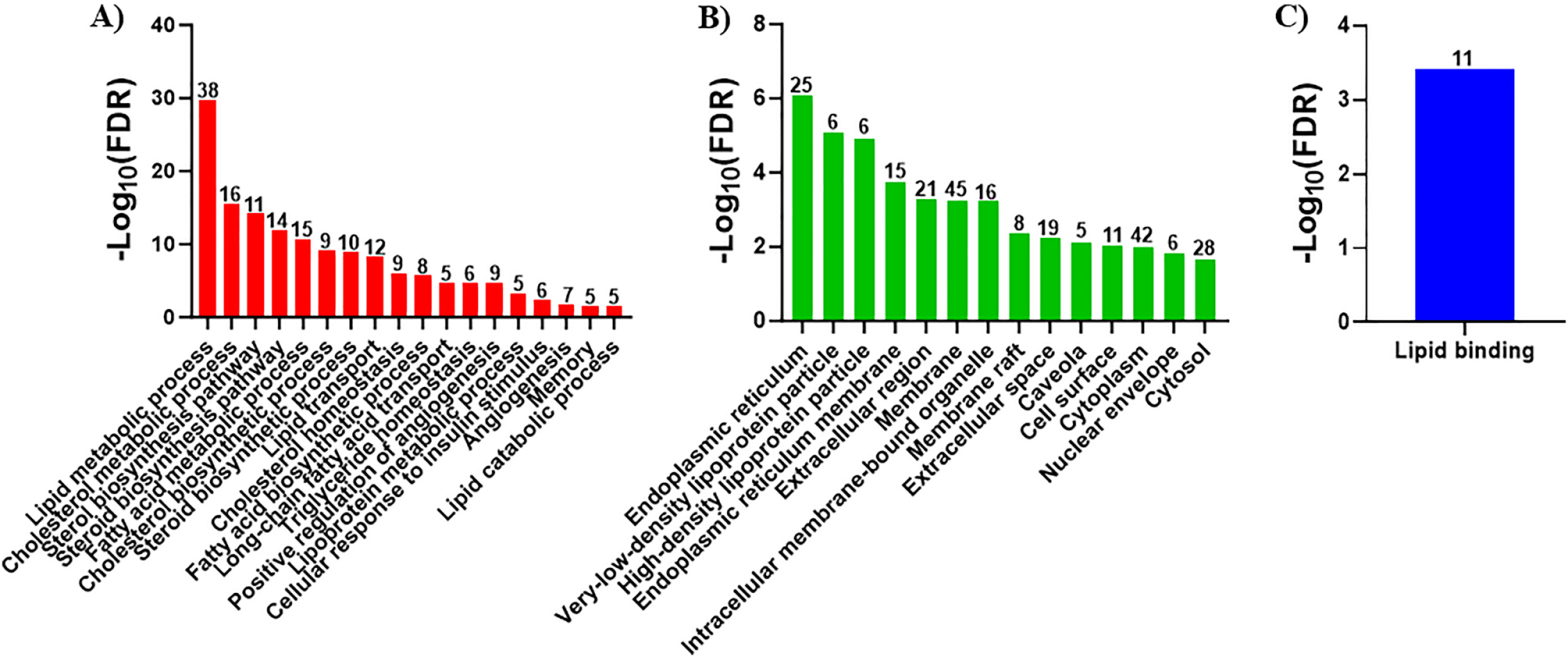
Mouse microglia GO enrichment analysis. Biological function analyses for the mouse microglial lipid metabolism DEG was performed using DAVID. Analyses were performed for biological process (BP) (**A**), cellular component (CC) (**B**), and molecular function (MF) (**C**). Pathways are shown in descending order based on − log_10_ FDR. The number of genes associated with each GO term is shown above each bar. Only GO terms with a gene count ≥ 5 and FDR < 0.05 were considered significant.

For mouse microglial lipid metabolism DEG, BP GO indicated the genes participated in lipid metabolic process, cholesterol metabolic process, sterol biosynthetic process, steroid biosynthetic process, fatty acid metabolic process, cholesterol biosynthetic process, steroid biosynthetic process, lipid transport, cholesterol homeostasis, fatty acid biosynthetic process, long-chain fatty acid transport, triglyceride homeostasis, positive regulation of angiogenesis, lipoprotein metabolic process, cellular response to insulin stimulus, angiogenesis, memory, and lipid catabolic process (Fig. 3A). CC GO indicated the mouse microglial lipid metabolism DEG were located in the endoplasmic reticulum, very-low-density lipoprotein particle, high-density lipoprotein particle, endoplasmic reticulum membrane, extracellular region, membrane, intracellular membrane-bound organelle, membrane raft, extracellular space, caveolae, cell surface, cytoplasm, nuclear envelope, and cytosol (Fig. 3B). MF GO indicated the mouse microglial lipid metabolism DEG were involved in lipid binding (Fig. 3C). A complete list of the GO, false discovery rates, and genes associated with each GO is shown in Supplementary Table S4.

**Figure 4:**
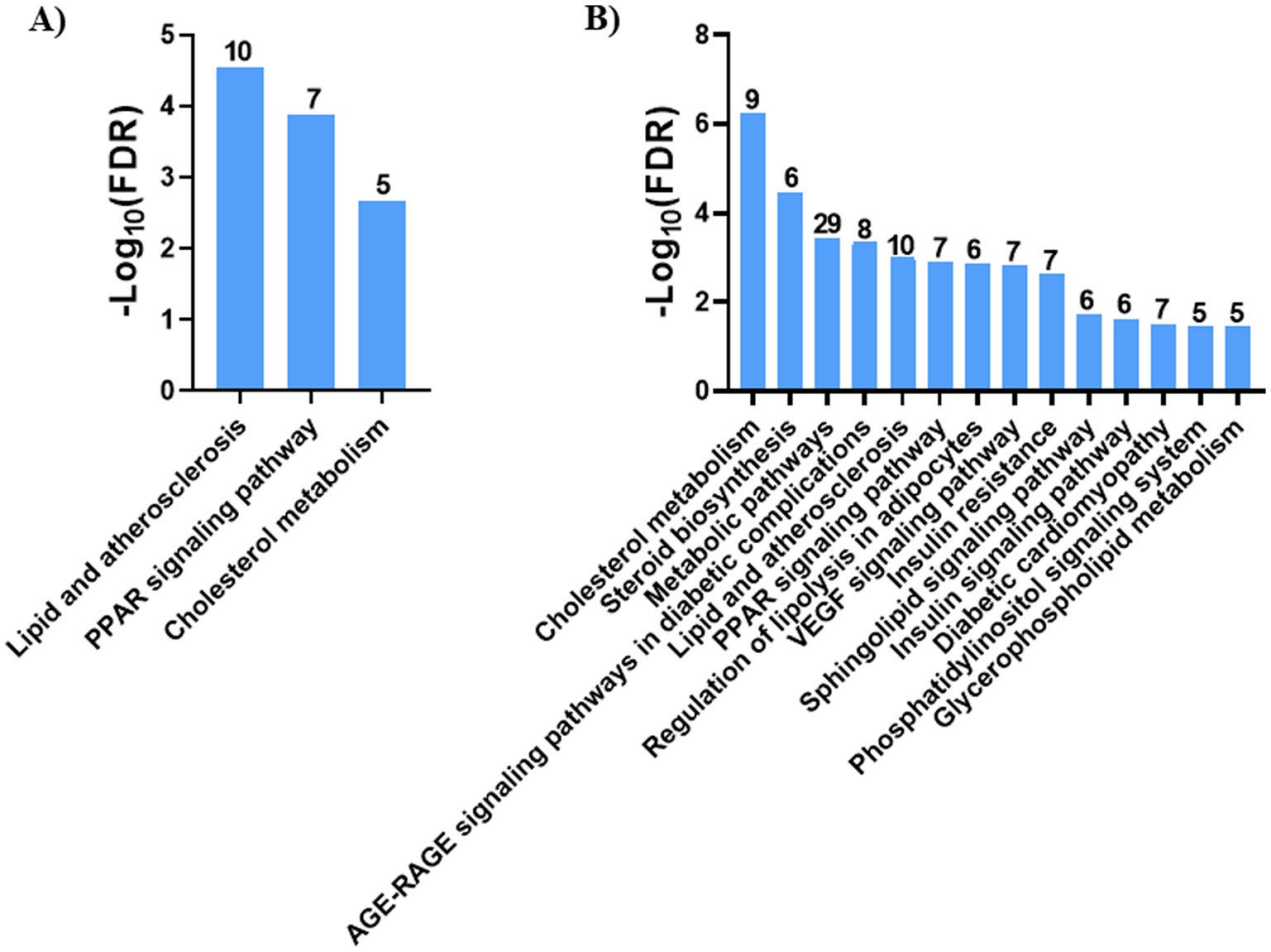
KEGG pathway enrichment analysis. KEGG pathway analysis was performed on the lipid metabolism DEG from the N9 microglia RNA-seq (**A**) and mouse microglia microarray (**B**) using DAVID. Pathways are shown in descending order based on − log_10_ FDR. The number of genes associated with each pathway is shown above each bar. Only pathways with a gene count ≥ 5 and FDR < 0.05 were considered significant.

KEGG analysis was performed on the N9 and mouse microglial lipid metabolism DEG. For N9 microglia, KEGG identified three pathways (lipid and atherosclerosis, PPAR signaling pathway, cholesterol metabolism) associated with the DEG (Fig. 4A). A complete list of the KEGG pathways, false discovery rates, and genes associated with each pathway is shown in Supplementary Table S5. For the mouse microglial lipid metabolism DEG, KEGG identified a total of 14 associated with the DEG (Fig. 4B). The pathways included cholesterol homeostasis, steroid biosynthesis, metabolic pathways, AGE-RAGE signaling pathway in diabetic complications, lipid and atherosclerosis, PPAR signaling pathway, regulation of lipolysis in adipocytes, VEGF signaling pathway, insulin resistance, sphingolipid signaling pathway, insulin signaling pathway, diabetic cardiomyopathy, phosphatidylinositol signaling system, glycerolphospholipid metabolism. A complete list of the KEGG pathways, false discovery rates, and genes associated with each pathway is shown in Supplementary Table S6.

**Figure 5:**
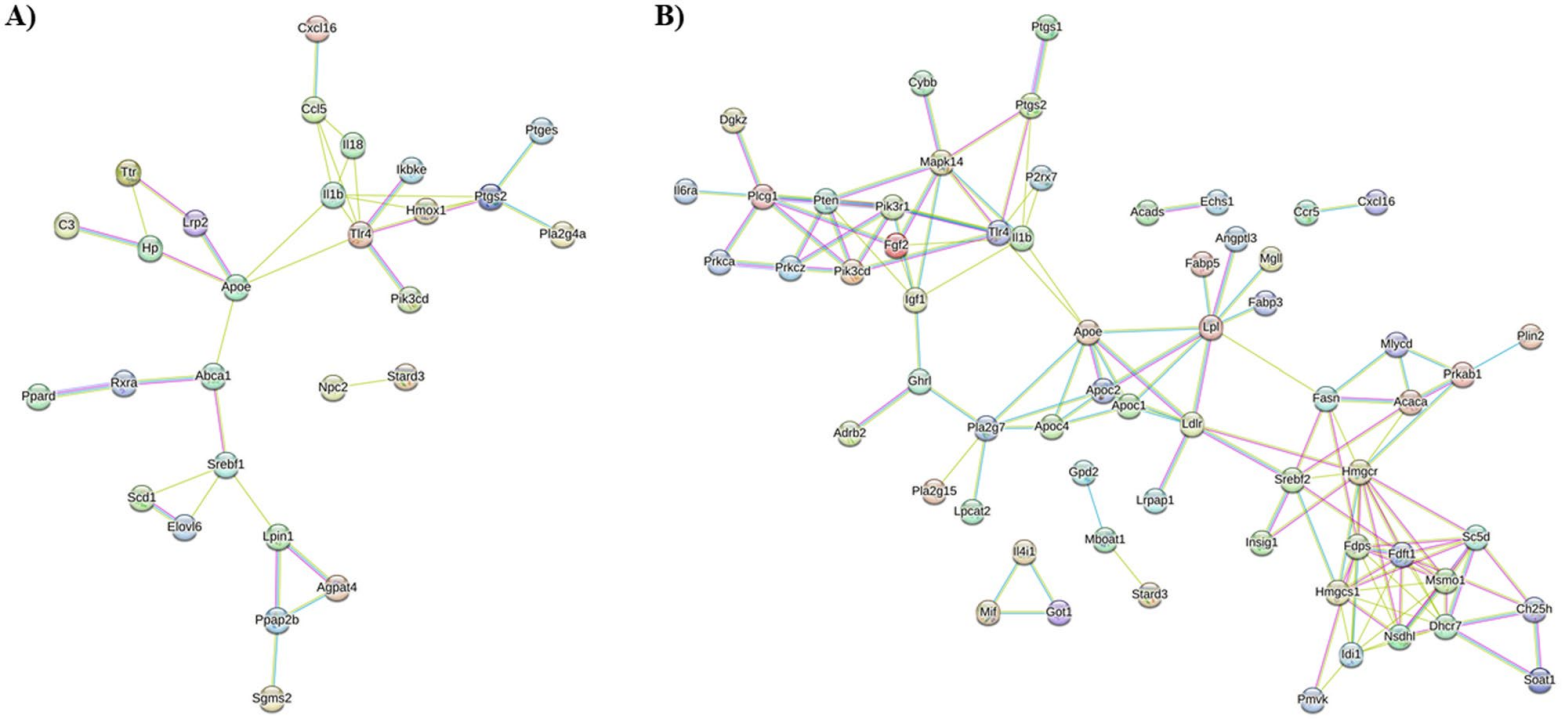
PPI analysis using STRING. STRING analysis was performed on the N9 (**A**) and mouse (**B**) microglial lipid metabolism DEG. For the analysis, text mining, experiments, and databases were chosen for active interaction sources, and a high value of 0.700 was selected as the minimum required interaction score. Line colors represent known interactions from curated databases (blue), experimentation (purple) and text mining (yellow). The proteins for which there were no connections to be mapped are not shown.

To further understand the interactions of the lipid metabolism genes, we performed protein–protein interaction (PPI) analysis on the N9 and mouse microglial DEG using STRING to identify known and predicted PPIs. Of the proteins encoded by the N9 microglial lipid metabolism DEG, 26 proteins clustered in a large network with two proteins forming a second, small cluster, and 24 proteins not clustering (Fig. 5A). For the mouse microglial lipid metabolism DEG, 52 proteins clustered in a large network, with 10 proteins forming four distinct smaller networks, and 24 proteins not clustering (Fig. 5B). The results suggest that the given proteins were highly enriched for both the N9 and mouse datasets (*p* < 1 × 10^–16^) indicating that the interactions were significantly more than those expected for a random collection of input genes, and these PPI networks could be significantly altered in lipid metabolism and AD-associated microglial neuroinflammation.

### Lipid metabolism gene targets for therapeutic drugs

**Figure 6:**
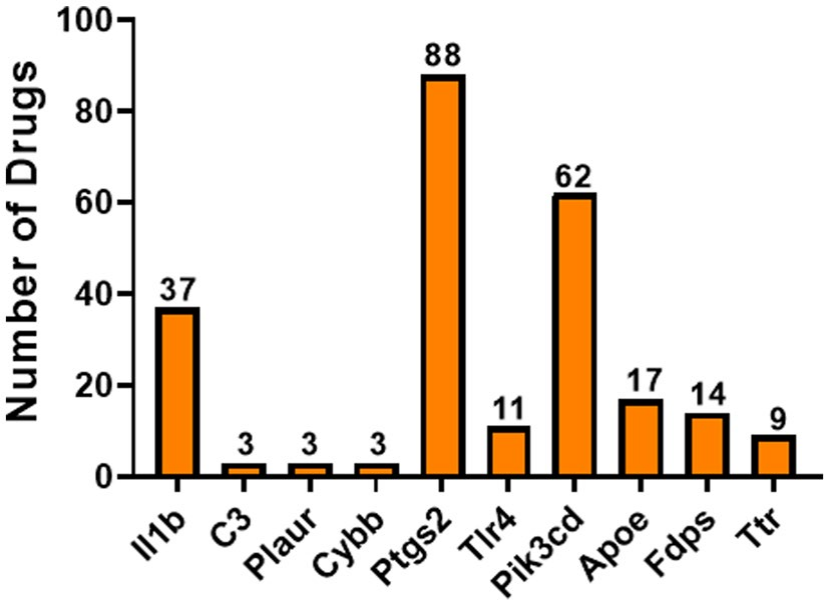
Gene–drug interactions. Interactions between therapeutic drugs and the 16 lipid metabolism DEG common to both datasets. Genes identified with drug interactions are shown and the number of drugs associated with each gene is shown above each bar.

In order to determine lipid metabolism gene targets for therapeutic drugs, we performed gene–drug interactions in the drug–gene interaction database (DGIdb)^23,24^ using the 16 lipid metabolism DEG common to both datasets (Table 1). A total of ten lipid metabolism genes had interactions with therapeutic drugs (Fig. 6). Prostaglandin-endoperoxide synthase 2 (*Ptgs2*) had the most interactions (88 drugs) with most of the drugs identified used to treat arthritis, pain, fever, and inflammation. The DGIdb gene–drug interaction tool also identified the hypertension drugs reserpine and atenolol as targets for *Ptgs2*. Transthyretin (*Ttr*) (the most down-regulated gene in both datasets), had nine drug interactions identified with several of the drugs used for transthyretin-mediated amyloidosis (tafamidis, tafamidis meglumine, inotersen, patisiran). Interleukin 1 beta (*Il1b*) (highly up-regulated in both datasets) had 37 drugs interactions with the majority of the drugs used to treat arthritis and inflammation. Finally, several other drugs were also identified as potentially interacting with our genes of interest including those targeting hypertension (verapamil, nicardapine), cholesterol (pravastatin), bipolar disorder (lithium), and microglial activation inhibition (TT 301) drugs for *Il1b*. A complete list of the lipid metabolism DEG and their associated drugs is shown in Supplementary Table S7.

### *Cybb* network analysis

**Figure 7:**
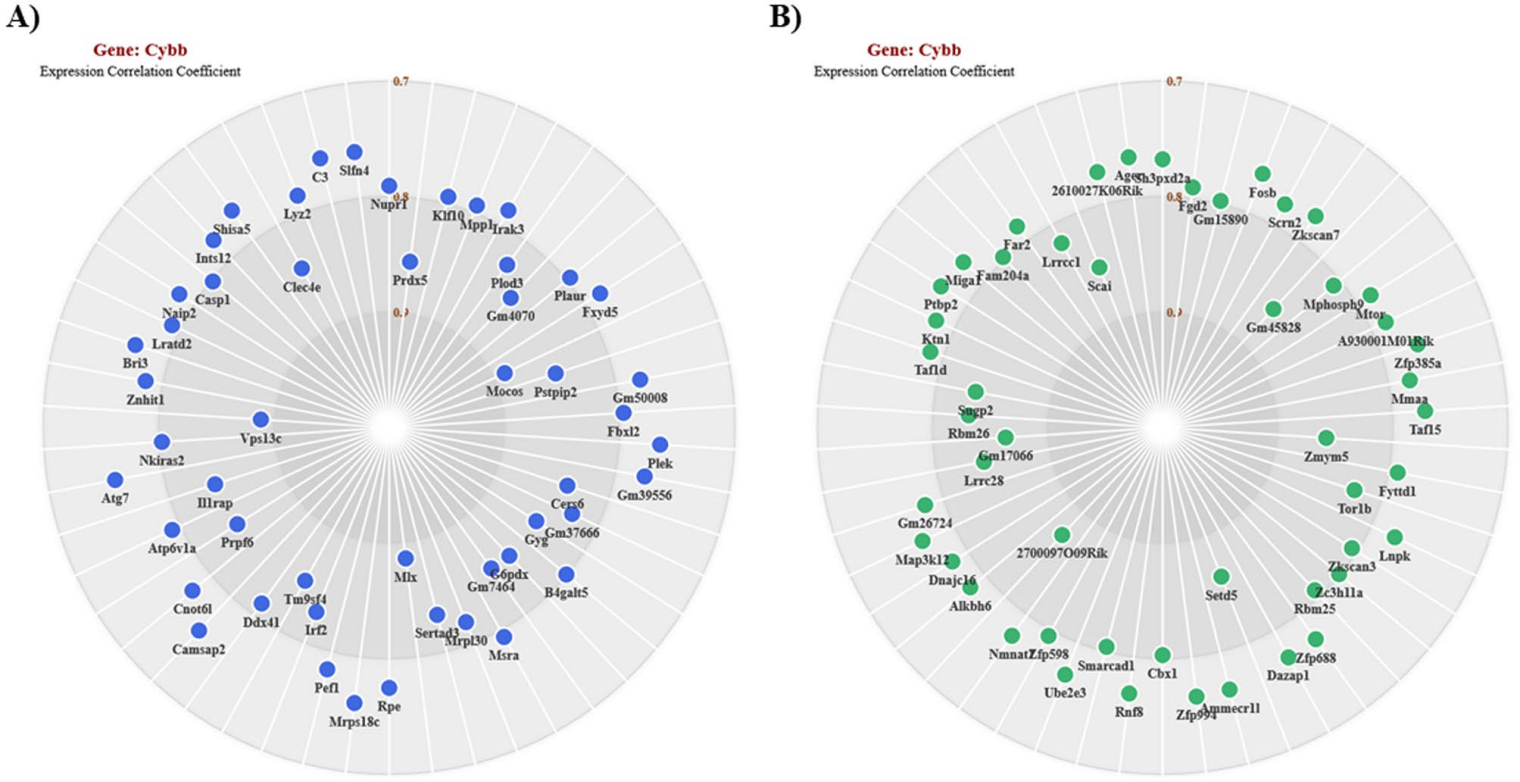
*Cybb* expression network analysis. Gene constellations for *Cybb* were created using ImmGen. **(A)** Positive expression correlation of genes to *Cybb*. **(B)** Negative expression correlation of genes to *Cybb*.

Since *Cybb* was altered in both datasets, involved in several pathways at the intersection of diabetes and cardiovascular disease, and was the target of several drugs, expression network analysis was performed to identify genes positively and negatively correlated with *Cybb*. In the positive correlation map, several genes involved in lipid metabolism and inflammation including *Plaur* (0.796), *Irak3* (0.785), *Prdx5* (0.855), *Casp1* (0.802), and *Naip2* (0.784) displayed a positive relationship with *Cybb* (Fig. 7A). In the negative correlation map, *Mtor* (− 0.786), a gene that controls most metabolic processes in response to nutrients, displayed a negative relationship with *Cybb* (Fig. 7B). Together, these data suggest *Cybb* could be a promising target for future research investigating gene networks for AD treatment strategies.

## Discussion

Investigating N9 and mouse microglial gene expression datasets, we identified several lipid metabolism genes which may be important in AD development. Diabetes, hypertension, hypercholesterolemia, and hypertriglyceridemia have all been identified as major risk factors of AD, but the mechanisms between these metabolic syndromes and AD remains unclear^8,20,25^. Statins are the most commonly used drugs for treating lipid disorders and are effective in reducing cholesterol and triglyceride levels. Conflicting results are reported when accessing statins specifically for AD, as several studies suggest statins slow the progression of AD^26–29^ while others suggest statins provide no protective benefit against AD^30–32^. Overall, the relationship between statins and cognitive function remains unclear, and further investigation is needed to determine if statins are an appropriate therapeutic strategy for AD.

Recently, several studies have highlighted the importance of microglial lipid metabolism in AD. Recent review articles provide a detailed overview of microglial lipid metabolism in regards to altered brain function in AD^9,10^. In a single-cell RNA sequencing (scRNA-seq) study, Keren-Shaul et al*.* used the 5XFAD mouse model of AD to demonstrate a preference for lipids as a fuel source during the increased metabolic energy demands of activated microglia^33^. Furthermore, their study identified a unique subset of microglia, disease associated microglia (DAM), which have a unique transcriptional profile associated with several lipid and metabolism genes, such as triggering receptor expressed on myeloid cells 2 (*Trem2*), lipoprotein lipase (*Lpl*), and *ApoE*^33^. In another study, Krasemann et al*.* described a distinct gene expression pattern associated with a TREM2- and APOE-dependent response by microglia to tissue damage in the brain^34^. In human AD, and the APP-PS1 mouse model of AD, they demonstrated the APOE pathway, driven by TREM2, mediated a switch in microglial phenotypes from homeostatic to neurodegenerative, indicating activation of the TREM2-APOE pathway leads to the inability of microglia to regulate brain homeostasis^34^. Further understanding of microglia-lipid interactions, along with advances in lipidomics technologies^35,36^, could aide in the development of new treatment regimens for AD.

Neuroinflammation drives AD pathogenesis by exacerbating both amyloid and tau pathologies^37^. Hypercholesterolemia has been linked to cognitive dysfunction accelerated by neuroinflammation in mice^38^ and rats^39^ fed a high fat diet. In these studies, activated microglia and astrocytes in the hippocampus released several proinflammatory cytokines, including IL-1β^38,39^. IL-1β was highly up-regulated in both our datasets, and is a vital component of lipid metabolism through the regulation of *Lpl*^40,41^. Activation of the nod-like receptor family pyrin domain containing 3 (NLRP3) inflammasome is a major source of IL-1β, and the NLRP3 inflammasome induces chronic neuroinflammation that significantly increases AD pathology^42^. Recently, several studies have used the ketone metabolite, β-hydroxybutyrate, to block activation of the NLRP3 inflammasome as a therapeutic strategy for general inflammatory disease^43^ and AD^44^. The gene-drug analysis performed in our study, identified 37 drugs targeting IL-1β. These data suggest therapeutic agents that block activation of the NLRP3 inflammasome and IL-1β as promising treatment strategies for inflammatory diseases, including AD.

Cytochrome b-245, beta polypeptide (*Cybb*) is a subunit of nicotinamide adenine dinucleotide phosphate (NADPH) oxidase that produces reactive oxygen species (ROS) which mediate microglial inflammatory responses^45^. If not properly regulated, ROS production by microglia leads to neuron damage, dysfunction, and death^46^. In our study, *Cybb* was up-regulated in both datasets. Network analysis revealed a positive correlation between *Cybb* and the inflammatory genes *Casp1* and *Naip2*. BP GO analysis identified *Cybb* in the positive regulation of angiogenesis, and KEGG pathway analysis identified *Cybb* in the lipid and atherosclerosis, AGE-RAGE signaling pathways in diabetic complications, and diabetic cardiomyopathy pathways. Additionally, our gene-drug interaction analysis identified three compounds that target *Cybb*. Taken together, these data suggest *Cybb* is at the intersection of cardiovascular disease, diabetes, and neuroinflammation, making it an attractive potential target for further investigation.

The identification of enriched lipid metabolism DEGs in cellular compartments (GO CC) such as the endoplasmic reticulum (ER), caveolae, and Golgi apparatus, provides further insight into the role of lipid metabolism DEGs in microglial-mediated neuroinflammation in AD. Increasing evidence suggests sustained ER stress contributes to neuron damage, microglial polarization, and altered inflammatory responses, particularly in LPS-stimulated cells^47–49^. Furthermore, ER stress is shown to activate microglia^50^ and inhibiting ER stress displays a neuroprotective effect in LPS-stimulated BV2 microglia^51^. The Golgi apparatus is essential for the synthesis and modification of proteins and lipids, and Golgi fragmentation has been observed in AD^52^. Golgi fragmentation is suggested to promote neuronal ion channel damage and the accumulation of tau and Aβ^53^. Specialized regions of plasma membranes, such as caveolae, modulate reactive oxygen species (ROS) producing systems^54^. Activated microglia produce ROS, along with many other proinflammatory molecules implicated in AD pathogenesis^2^. In our study, a large number of lipid metabolism DEGs in N9 and mouse microglia were found in the ER and Golgi apparatus, including *ApoE* and *Cybb*. Additionally, several mouse microglial lipid metabolism DEGs localized in the caveolae, including low density lipoprotein receptor (*Ldlr*) which is a primary metabolic receptor responsible for ApoE lipoprotein clearance^55,56^. In the study by Shi et al*.*, a neuroprotective role for Ldlr was described as Ldlr overexpression attenuated tau pathology through preservation of myelin, inhibiting microglial activation, and reducing *ApoE* levels, suggesting drug discovery directed towards increasing Ldlr levels as a treatment option for AD and other tauopathies^57^.

One of the primary limitations of our study is that only murine microglia were used. We examined several publicly available human AD transcriptome datasets in an attempt to correlate our findings to AD in humans. These datasets indicated most lipid metabolism genes identified in our study were differentially regulated in human datasets as well. The issue, however, was that even though the human datasets analyzed showed a large number of overall genes altered in AD brains versus non-AD controls, the level of differential gene expression was not nearly as robust as seen in our mouse and cell culture transcriptome datasets. Gene expression analysis of human brain tissue and cells is certainly a valuable tool for investigating AD in humans. Brain tissue, however, can only be collected postmortem, and peripheral tissues and blood, which can easily be collected from patients, may not express brain proteins that are central to AD development and progression^58^. RNA stability in human samples is also a concern as confounding factors, like differences in postmortem interval, have been shown to contribute to inaccuracies in human brain transcriptomic data^59^. Furthermore, comparing the mouse and human AD transcriptome remains unclear, as some studies indicated the transcriptomes are similar^60,61^ while others suggest they are different^62,63^. A strength of our study is that N9 cell culture and primary microglia from mice were used. By comparing these datasets, we were able to determine genes which are part of the neuroinflammatory aspect of AD development since lipopolysaccharide (LPS) treated N9 microglia would primarily represent the general neuroinflammation process. Overall, these data offer several microglial gene target–drug interactions for initial validation in mouse models of AD.

In summary, our transcriptome profiling data offers a useful resource to the field for understanding the inflammatory roles of microglial lipid metabolism genes in AD. Furthermore, our data identified drugs for microglial molecular targets for future investigation to attenuate/eliminate the pathological progression of AD.

## Methods

### Datasets

The N9 microglial RNA-seq dataset was published previously by our group (GSE183038)^64^. Briefly, immortalized murine N9 microglia were routinely cultured^65^ and seeded at a cell density of 250,000 cells/well in a 24-well tissue culture plate. Cells were stimulated with LPS (1 µg/ml) from *Escherichia coli* O111:B4 (InvivoGen) for 6 h. RNA was extracted using an RNeasy Plus Mini Kit (Qiagen, Cat. No. 74134). Quality and quantity of RNA was assessed using an Agilent 2100 Bioanalyzer (Agilent Technologies) and a Nanodrop spectrophotometer (Thermo Scientific). All samples had an RNA integrity number (RIN) of 9.7 or higher. RNA library preparation and transcriptome sequencing were performed by Novogene using the Illumina NovaSeq 6000 Sequencing System. Genes with FDR-adjusted *p*-values < 0.5 and log_2_FC > 0.5 were considered differentially expressed.

The mouse microarray has been published in a previous study^21^ and the publicly available dataset (GSE65067) was used. Briefly, microglia from female 8 month old wild-type (n = 3) and 5XFAD (n = 5) mice (The Jackson Laboratory) were FACS-sorted directly into RTL-plus lysis buffer. RNA extraction from microglia was performed using an RNeasy Plus Micro Kit (Qiagen, Cat. No. 74034). Microarray hybridization (Affymetrix MoGene 1.0 ST array) and data processing were performed at the Washington University Genome Center. Genes with *p*-values < 0.05 and log_2_FC > 0.5 were considered differentially expressed.

### Gene analyses

In order to identify lipid metabolism genes, the datasets were searched for the terms “lipid”, “lipoprotein” and “cholesterol”. Additionally, datasets were searched for lipid metabolism genes from lipoprotein signaling, cholesterol metabolism, and lipid metabolism pathway analysis gene lists (Supplementary Table S8).

Lipid metabolism genes found to be differentially expressed were selected for biological function analysis. The gene list was uploaded into the Database for Annotation, Visualization and Integrated Discovery (DAVID, v. 6.8)^66,67^ for GO and KEGG pathway analysis. KEGG pathways and GO BP, CC, and MF with gene counts ≥ 5 and FDR < 0.05 were considered significant. Additionally, PPI analysis was performed to identify interactions of the selected proteins based on their gene IDs using the STRING database^68^. For the analysis, text mining, experiments, and databases were chosen for active interaction sources using the high confidence (0.700) threshold setting.

Scatterplots were created using Prism 9.0.0 (GraphPad). Venn diagrams demonstrating overlap in lipid metabolism DEG amongst the datasets were generated using InteractiVenn^69^. Gene constellations identifying genes in the *Cybb* regulatory network were created with ImmGen^70^ using the “myeloid cells” reference populations option. Gene-drug interactions of the common lipid metabolism DEG were identified using DGIdb^23,24^ using the default settings.

### Supplementary Information

Supplementary Tables.

## Data Availability

The datasets generated and/or analyzed during the current study are available in the Gene Expression Omnibus (GEO) repository, GSE183038 and GSE65067.
